# Effects of water availability on a forestry pathosystem: fungal strain-specific variation in disease severity

**DOI:** 10.1038/s41598-017-13512-y

**Published:** 2017-10-18

**Authors:** Riikka Linnakoski, Junko Sugano, Samuli Junttila, Pertti Pulkkinen, Fred O. Asiegbu, Kristian M. Forbes

**Affiliations:** 1 Department of Forest Sciences, University of Helsinki, FI-00014 Helsinki, Finland; 2 Centre of Excellence in Laser Scanning Research, Finnish Geospatial Research Institute FGI, FI-02430 Masala, Finland; 3 Natural Resources Institute Finland (Luke), FI-12600 Läyliäinen, Finland; 4 Department of Virology, University of Helsinki, FI-00290 Helsinki, Finland

## Abstract

Norway spruce is one of the most important commercial forestry species in Europe, and is commonly infected by the bark beetle-vectored necrotrophic fungus, *Endoconidiophora polonica*. Spruce trees display a restricted capacity to respond to environmental perturbations, and we hypothesized that water limitation will increase disease severity in this pathosystem. To test this prediction, 737 seedlings were randomized to high (W+) or low (W−) water availability treatment groups, and experimentally inoculated with one of three *E. polonica* strains or mock-inoculated. Seedling mortality was monitored throughout an annual growing season, and total seedling growth and lesion length indices were measured at the experiment conclusion. Seedling growth was greater in the W+ than W− treatment group, demonstrating limitation due to water availability. For seedlings infected with two of the fungal strains, no differences in disease severity occurred in response to water availability. For the third fungal strain, however, greater disease severity (mortality and lesion lengths) occurred in W− than W+ seedlings. While the co-circulation in nature of multiple *E. polonica* strains of varying virulence is known, this is the first experimental evidence that water availability can alter strain-specific disease severity.

## Introduction

The frequency and intensity of droughts and floods is expected to increase due to climate change, with substantial supportive evidence already accumulated^1^. In addition to their direct negative impacts on plant health, extreme and persistent weather events have the potential to impair plant responses to other environmental stressors, such as pathogen infections^2–4^. Largely due to changes in their geographical distributions and an increased evolutionary capacity often conferred by a warmer environment, pests and pathogens represent one of the greatest threats to global forest health under climate change^2–5^. In order to prepare for and potentially mitigate these effects, it is necessary to empirically evaluate how economically important forestry pathosystems will respond to such environmental perturbations.

Forestry is a key economic industry in northern Europe. For example, in Finland it comprises approximately 20% of national export revenue, and 4.2% of all gross domestic product (GDP)^6^. Norway spruce (*Picea abies*) is one of the most frequently employed commercial forestry tree species in the region. However, this boreal-adapted species is sensitive to abiotic and biotic disturbances, including acute soil water deficit, wind and snow damage, and pest and pathogen attack^7–9^. Boreal ecosystems are expected to be some of the most severely impacted regions by climate change^1,10^, which thus has the potential to influence forestry output and practices.

A necrotrophic pathogen of increasing concern to Norway spruce is *Endoconidiophora polonica*, which has been demonstrated capable of killing mature trees^11–13^. This fungus is commonly vectored by the Eurasian spruce bark beetle (*Ips typographus*); the most important pest of mature *P. abies* trees in Europe^14^. Warming temperatures and environmental extremes associated with climate change (such as wind and snow storms, which create tree damage that facilitates beetle colonization) are likely to increase bark beetle abundance in boreal regions through more favorable breeding conditions^15^. Moreover, drought is considered one of the most important factors impairing the resistance of Norway spruce to *I. typographus* infections^16^. Based on recent evidence, low-level drought stress increases beetle attacks, but trees that suffer extreme drought stress are less likely to be infested by the beetles^17^.

In addition to their direct negative effects on tree health, increasing evidence indicates that fungal infections are critical for bark beetle success, as *E. polonica* plays an important role in overcoming *P. abies* defense systems in living trees^18–21^. As such, it is likely that low-level and persistent physiological tree stressors, such as drought, will interact with less-frequent but more extreme environmental perturbations, such as storms, to exacerbate fungal-induced damage to boreal spruce forests under climate change. However, previous research has identified complex and difficult to anticipate interactions between climate-induced tree stress and pathogen infections^4,22^, with some evidence that persistent water restriction could also increase tree resistance to *E. polonica* ^21–23,24]^. It is therefore necessary to empirically evaluate responses of this pathosystem to water restriction in a rigorous experimental setting.

Here we report on a large *in vivo* experiment, conducted in an open greenhouse in southern Finland, to evaluate the effects of water availability on *E. polonica* infected Norway spruce seedlings during an annual growing season. Seedlings have been previously demonstrated as an effective model for larger tree health in this pathosystem^12,13^. A total of 737 seedlings were randomized to high (W+) or low (W−) water availability treatment groups, and inoculated with one of three fungal strains (designated F3 - F5^25^) or mock-inoculated. Seedlings were monitored for mortality throughout an annual growing season, and total growth and lesion length indices were measured at the experiment conclusion. Due to the low tolerance of Norway spruce to acute water deficiency we hypothesized that reduced water availability will lead to greater disease severity when compared to seedlings with high water availability.

## Results

**Figure 1:**
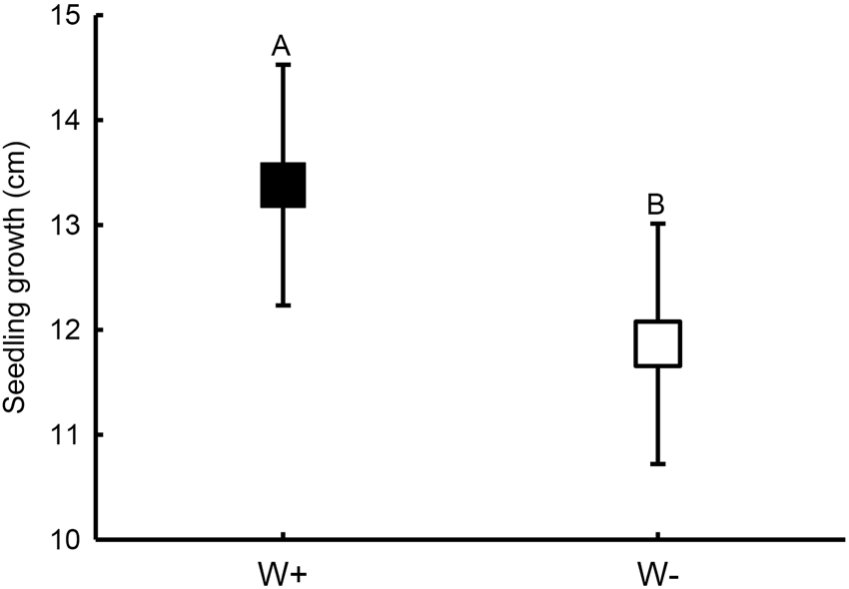
Estimated marginal means (95% CI) of seedling growth during the experiment. Different letters above error bars represent a statistically significant (P < 0.05) difference between treatment groups.

**Figure 2:**
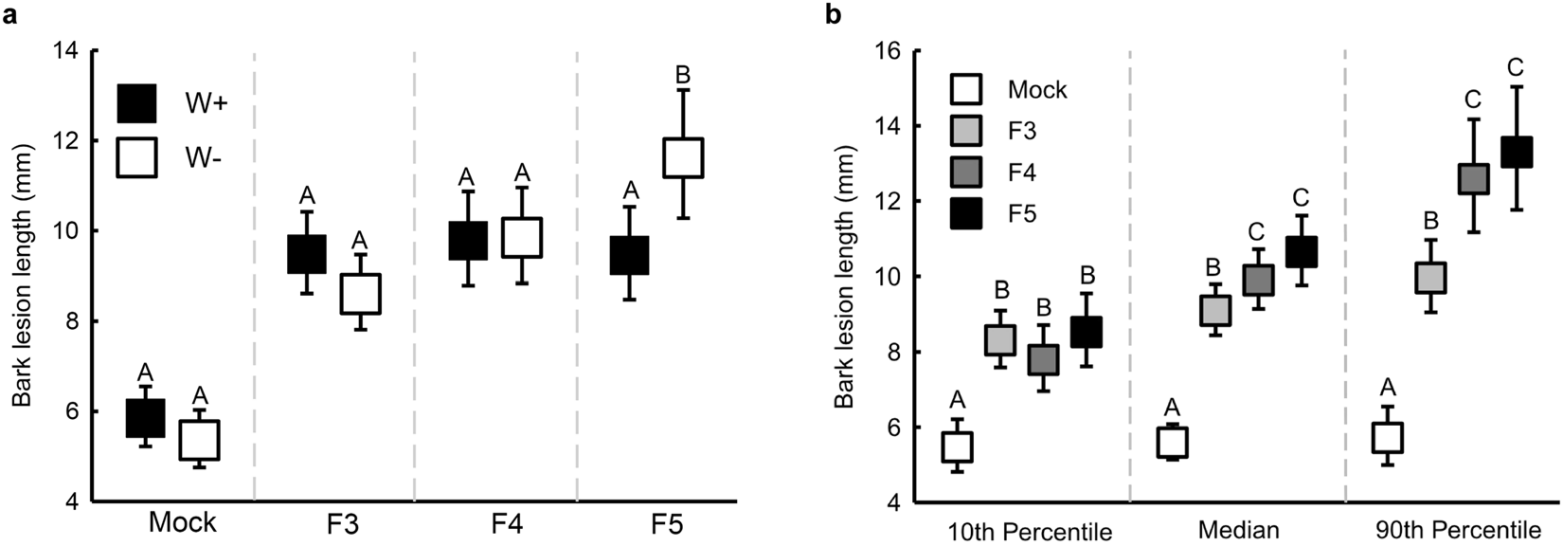
Estimated marginal means of bark lesion lengths (95% CI) at the experiment conclusion in relation to (**a**) water availability treatment group. Different letters above error bars represent statistically significant within-strain differences between treatment groups, and (**b**) seedling height at the beginning of the experiment (which is shown along the X-axis). Different letters above error bars represent statistically significant differences among strains.

Seedling growth was greater in the high water availability treatment (W+) group than the low water availability (W−) treatment group (Table 1, Fig. 1), and was not affected by the fungal infections or length of the sapwood lesion. Bark lesion length was significantly influenced by the interaction between water availability treatment group and fungal strain (Fig. 2a); seedlings infected with fungus F5 displayed longer lesions under the W− treatment than W+ treatment. Bark lesion lengths did not vary between water treatment groups for seedlings infected with the other two fungal strains. Bark lesion lengths also varied among fungal strains due to the height of seedlings at the beginning of the experiment (Table 1); in the shortest seedlings (10^th^ percentile) no variation occurred among fungal strains, while in taller seedlings (median and 90^th^ percentile) lesions were longer due to infections with fungal strains F4 and F5 than F3, and did not vary between F4 and F5 (Fig. 2b). Bark lesion lengths were larger across all fungal strains than with the mock inoculations. The same pattern of effects as seen for the bark lesion length occurred for the sapwood lesion length (Table 1).

**Table 1:** Most parsimonious models to explain response variables. Initial models included all interactions between sources of variation.

Response	Source of variation	Num. d.f	Den. d.f	F	P
Seedling growth	Sapwood lesion	1	484	1.17	0.28
	Fungal strain	3	488	0.47	0.71
	**Water treatment**	**1**	**29**	**16.15**	**0.0004**
Bark lesion length	Beginning height	1	459	40.30	<0.0001
	Fungal strain	3	477	2.49	0.0596
	Water treatment	1	32	0.28	0.60
	**Height × Strain**	**3**	**478**	**5.40**	**0.0012**
	**Treatment × Strain**	**3**	**476**	**5.41**	**0.0011**
Sapwood lesion length	Beginning height	1	451	43.49	<0.0001
	Fungal strain	3	476	2.65	0.0481
	Water treatment	1	33	0.58	0.45
	**Height × Strain**	**3**	**477**	**5.79**	**0.0007**
	**Treatment × Strain**	**3**	**472**	**5.16**	**0.0016**
Mortality–1^st^ interval	**Fungal Strain**	**2**	**601**	**17.90**	**<0.0001**
	Water treatment	1	601	0.41	0.52
Mortality–2^nd^ interval	Fungal strain	2	430	8.19	0.0003
	Water treatment	1	430	0.50	0.48
	**Treatment × Strain**	**2**	**430**	**3.26**	**0.0394**
Box weight	Monitoring occasion	6	25	37.52	<0.0001
	Water treatment	1	30	370.37	<0.0001
	**Treatment × Occasion**	**6**	**25**	**70.97**	**<0.0001**

Almost all seedling mortality occurred during the first two monitoring intervals, which were analyzed in separate regression models. Highest order significant effects (P < 0.05) are bolded.

**Figure 3:**
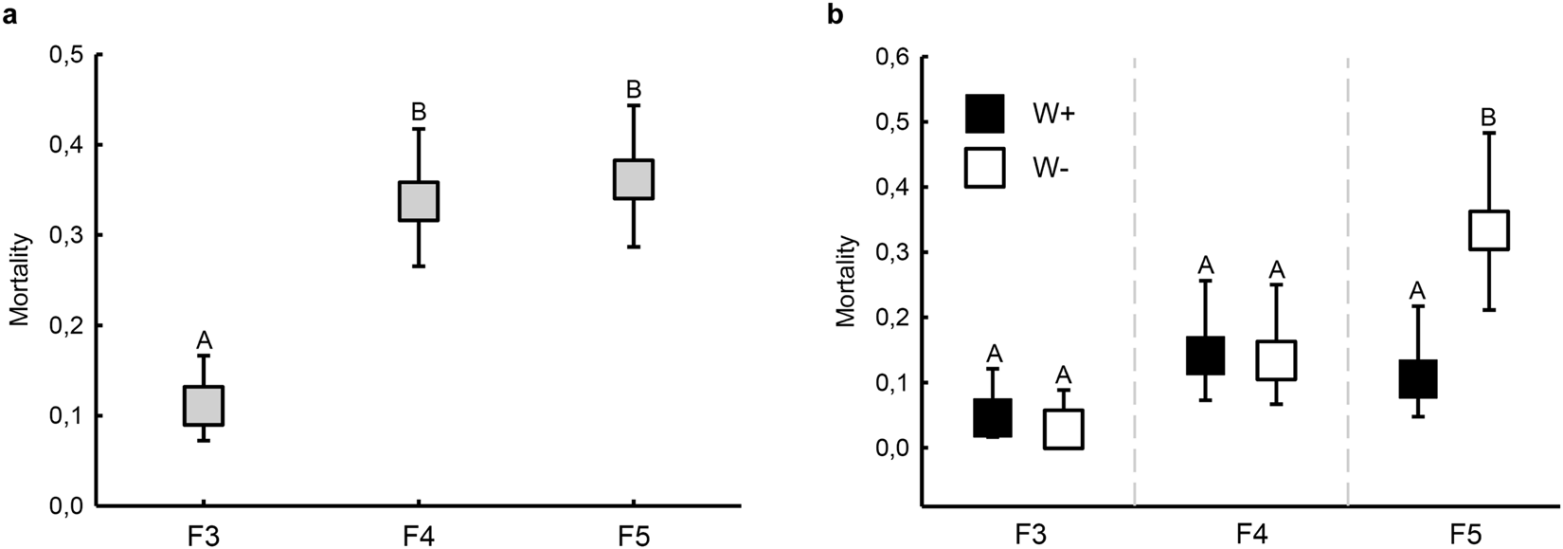
Estimated marginal means of seedling mortality (95% CI) (**a**) during the first month of the experiment (no difference occurred between treatment groups). Different letters above error bars represent statistically significant differences between strains, and (**b**) during the second month of the experiment. Different letters above error bars represent statistically significant within-strain differences between treatment groups.

In total, 235 seedlings died during the experiment, with 97% of mortality occurring during the initial two months (170 seedlings died during the first monitoring interval, and 58 during the second). 52% of total mortality occurred in the W− treatment group. During the first month of the experiment, mortality rates varied between fungal strains (Table 1, Fig. 3a), but were not influenced by the water availability treatments; mortality was greater with fungal strains F4 and F5 than F3, but did not differ between F4 and F5. The same strain-specific differences occurred during the second monitoring occasion. However, during this time, mortality was also greater in W− than W+ seedlings that were inoculated with fungus F5 (Table 1, Fig. 3b). Only one mock-inoculated seedling died during the experiment (W+ treatment group).

**Figure 4:**
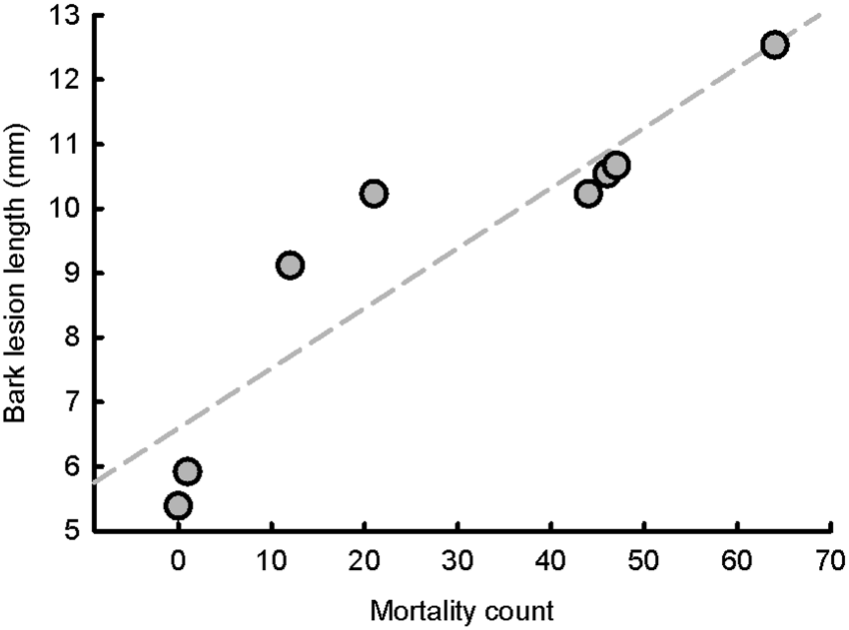
Relationship between bark lesion length and seedling mortality counts for each treatment group/fungal strain combination.

Bark and sapwood lesion lengths were strongly correlated with each other (r = 0.999, P < 0.0001) and each was also correlated with seedling mortality count (Bark lesion r = 0.91, P < 0.0017, Fig. 4; Sapwood lesion r = 0.92, P < 0.0011). Box weights did not vary between treatment groups at baseline, but were thereafter significantly greater in the W+ than the W− treatment group until the experiment conclusion (Table 1). *Endoconidiophora polonica* was successfully re-isolated from 77 of the 90 (85.6%) tested seedlings at the experiment conclusion.

## Discussion

Contrary to our broad expectation, disease severity was not universally greater for water restricted, fungus infected seedlings. Instead we found that the effects of water availability on infected seedlings varied among fungal strains. While all three fungal strains were pathogenic to seedlings - causing greater disease severity than mock-inoculations - no differences due to water availability were observed for two of the fungal strains (F3 and F4). For the third fungal strain (F5), greater disease severity, demonstrated through both larger lesion lengths and higher mortality rates, occurred in low water availability seedlings than those with high water availability. While the co-circulation in nature of multiple *E. polonica* strains of varying virulence has been previously established^12,18,26^, these results are the first experimental evidence that water availability can alter strain-specific virulence in this economically important forestry pathosystem.

Seedling growth was significantly greater under the high than low water availability treatment, demonstrating that the applied water restrictions led to reduced investment of nutrient and carbohydrate resources into primary metabolism/the establishment of above-ground tissue, as can be expected under a natural drought scenario^9^. This assumption is also supported by the differences in box weights between treatment groups. Soil water availability is one of the best documented factors affecting tree growth^9,27^. Since no seedling mortality occurred in the mock-inoculated W− seedlings, we conclude that the applied water restriction constituted a persistent, low-level stress in line with our manipulation objective.

Consistent with our previous experiment investigating temperature and CO_2_ effects on this pathosystem^25^, the vast majority of mortality occurred during the initial two months following experimental inoculations. As mortality differences between treatment groups occurred only during the second month of the experiment (see Fig. 3), water restriction was able to prolong the risk period for seedlings infected with fungus F5. Prolonged water stress is likely to compromise seedling resistance to this particularly aggressive fungal strain. However, further research addressing indices of resistance is required to validate this assumption. Equally interesting is that no such effect occurred for the other two fungal strains, demonstrating that seedlings with low water availability retained a certain level of defence capacity.

Pathogenic fungal infections induce defensive responses in trees (and seedlings) that aim to inhibit infection and promote wound healing^28^. Defenses are resource costly for hosts, and therefore often traded-off with other physiological processes, such as growth^29,30^. No growth differences occurred between fungal inoculated and mock-inoculated seedlings (nor was growth influenced by lesion length), suggesting that the inoculation wound alone required similar resource investment to that of a fungal infected wound; although since seedling lesion length was positively correlated with mortality and seedlings that died during the experiment were removed from the analyses of lesion lengths, potential differences are likely to be minimized. Consistent with healing and then redistribution of resources, all seedling inoculation wounds were covered with callus tissue at the end of the experiment, and seedling growth was much greater in the current experiment than during our previous shorter experiment, when many of the seedlings failed to grow at all^25^.

For the current experiment, seedlings were used in place of mature trees. This enabled us to employ large sample numbers (enabling strong statistical power), implement standardized manipulation treatments and also evaluate mortality, which is relatively rare for mature trees, all within a relatively short time period. While previous studies indicate that seedlings are an effective model for larger tree health in *E. polonica* – Norway spruce pathosystem^12,13^, subtle differences are likely, and we stress the importance of research on mature trees, which will ultimately be the most accurate reflection of *E. polonica* effects on spruce trees in nature.

The intensity and frequency of summer heat stress and drought associated with climate change is expected to increase in boreal regions^1,2^. Here we provide experimental evidence that reduced water availability can enhance disease severity, but significantly, that responses vary among fungal strains in the Norway spruce - *E. polonica* pathosystem. This study contributes to a limited body of rigorous experimental research on interactions between biotic agents and climate change-associated environmental perturbations, and will serve as foundation for future investigations. Further empirical and theoretical research, employing mature trees, and evaluating physiological and genetic measures of host resistance and pathogen virulence, is required to better understand, and ultimately predict, the impact of forest pathogens under climate change.

## Methods

### Plant and fungal material

Plant material consisted of 737 two-year old Norway spruce seedlings purchased from a nursery in Southern Finland (the same source stock used for commercial forestry). Seedlings were potted into plastic trays of 25 cells (each 8 × 8 × 9 cm), filled with fertilized peat, which were placed into larger plastic trays where excess water could accumulate and be absorbed later. They were acclimatized to the greenhouse conditions for two-weeks prior to the experiment, during which time they received tap water as required to maintain moist soil. No additional fertilization was given.

*E. polonica* strains were isolated from Eurasian spruce bark beetles in an outbreak region of Finland^31^. Fungal isolates were plated on 2% Malt Extract Agar (MEA) and grown at 25 °C for one week prior to the experimental inoculations. Three strains, identified by DNA sequencing of the internal transcribed spacer region (ITS)^31^ and with previously demonstrated pathogenicity^25^ [previously designated strains F3 - F5 (CBS 142281-142283), and referred to in the same way here for consistency], were employed for the current experiment.

### Experiment design

The experiment was conducted at the Haapastensyrjä field station of the Natural Resources Institute Finland (60°37′N, 24°26′E), from mid-May until the end of October, 2016 (encompassing the full annual growing season in the region). The 737 seedlings were randomly block-assigned by tray to either high (W+) or low (W−) water availability treatment groups, and experimentally inoculated with one of the three *E. polonica* fungal strains or mock-inoculated (mock-inoculations were capped at 100 seedlings; 50 per treatment group).

Seedling inoculations were made approximately mid-way up the stem on the first-year shoot. A sterile 5 mm cork borer was used to cut a bark flap, and an inoculum of the same size, cut from the actively growing outer zone of the culture plate, was placed onto the exposed sapwood surface. The inoculation site was then covered with the bark flap and sealed with Parafilm, which was removed approximately two weeks later. Mock inoculations were conducted following the same protocol, using sterile 2% MEA.

At the beginning of the experiment, each high water availability tray (25 seedlings) was given 2 L of water three times per week (Monday, Wednesday and Friday) and each low water availability tray was given 0.8 L of water twice per week (Monday and Friday). Water was poured over each seedling in the tray in an approximately even manner. After three-weeks, a small amount of stagnant water in the bottom of W+ trays revealed this amount to be too high, and the watering regimes were modified to 1.3 L × 3 (W+), and 1.0 L × 2 (W−), and also slightly altered the following week to 1.1 L × 3 (W+) and 1.1 L × 2 (W−). Water quantities were reduced one month later, in mid-July (W + 0.8 L × 3, W− 0.6 L × 2), and maintained at that level until early-October. They were then reduced again for the final two-weeks of the experiment (W + 0.6 L × 3, W− 0.3 L × 2). Overall, high water availability seedlings received slightly over double the water of low water availability seedlings. Seedling trays were rotated monthly to minimize potential variation in water requirements due to their positioning on the greenhouse benches.

Since seedling water intake varied with the ambient temperature (amongst other things), it was necessary to continuously monitor and adjust the watering schedule to maintain the dichotomy between high and low water availability treatments. Temperatures were monitored inside the greenhouse throughout the experiment using a DGT-Volmatic system (Odense, Denmark). During the experiment period, the average daily temperature (min–max) was 19.6 °C (6.7–38.5 °C) in May, 20.1 °C (7.7–34.3 °C) in June, 20.8 °C (11.1–35.0 °C) in July, 18.8 °C (9.4–34.1 °C) in August, 15.7 °C (6.6–30.5 °C) in September, and 9.4 °C (4.5–26.4 °C) in October.

### Data collection

Seedling height (nearest 0.5 cm) was measured at the beginning and end of the experiment, and used to calculate the total growth for each seedling during the experiment period (experiment end height - beginning height). Seedling mortality counts were monitored at monthly intervals throughout the experiment (five monitoring occasions). Seedling box weights were recorded at the same time as mortality observations, and also following the water regime change after three weeks. As the first weights were taken after watering, baseline weight was calculated for each box by subtracting the additional water weight. However, no measures of soil water content were collected.

Consistent with previous studies^12,25,32^, mortality was defined as discoloration of all seedling needles above the inoculation point. At the end of the experiment, the outer bark lesion length was measured with an electronic calliper (nearest 0.01 mm), and the bark was peeled back to expose the sapwood (xylem) lesion, which was also measured. The seedling stem was then cut in half slightly above the inoculation point to reveal the necrotic lesion depth. However, formation of callus tissue prevented meaningful assessment of the lesion depth into the sapwood.

To verify infections, fungi were re-isolated from 45 randomly selected inoculated seedlings from each treatment (in total 90 seedlings) at the end of the experiment. Samples were collected from the necrotic sapwood tissue and plated on 2% MEA. Plates were incubated at 25^o^C for up to 2 weeks, and inspected regularly for fungal growth. The presence of *E. polonica* was confirmed based on the morphological characteristics and microscopy.

### Data analysis

General linear mixed models were constructed to evaluate the effects of fungal infection and water availability treatments on seedling growth, and the bark and sapwood lesion length indices^33^. As these outcomes are inherently imprecise in dead wood, seedlings that died during the experiment were excluded from the analyses (235 seedlings). Data were log transformed (lesion lengths) and significant outliers (P < 0.05) were identified using Grubb’s test and removed. Initial fixed explanatory variables in the seedling growth model included water treatment group (categorical variable), fungal strain (categorical), sapwood lesion length (continuous), and all their interactions. Treatment group, fungal strain, height at the beginning of the experiment (continuous), and all interactions were set as initial fixed explanatory variables in the lesion length models. Seedling tray number (categorical) and position in the tray (three categories; rows/columns ranging from the outside to the interior to account for potential variation due to seedling position in the trays) were set as random factors in all models. Models were then sequentially reduced to achieve parsimony, whereby fixed effects interaction terms were removed if their exclusion did not increase Akaike information criterion (AIC) by >2 units. Model comparisons were made using the maximum likelihood (ML) method and final values were obtained using restricted maximum likelihood (REML). Model assumptions were visually checked from the residual distribution.

Generalized linear mixed models, with binary distributions and logit link functions, were used to evaluate the effects of fungal infections and water availability on seedling mortality. As the overwhelming majority of mortality occurred during the initial two months of the experiment (see results section), only data from the first two monitoring occasions were included. New mortalities for each monitoring interval were analyzed separately, and mock-inoculations were removed due to a lack of mortality (only one mock-inoculated seedling died during the experiment). Initial fixed explanatory variables were treatment group, fungal strain, and their interaction, and seedling tray number and position in the tray were again set as random factors.

Pearson correlation tests were used to assess the relationship between seedling mortality counts and the lesion lengths [comparing the seedling mortality count to the average lesion length (excluding mortalities) for each treatment group/fungal strain combination]. Outliers (P < 0.05) were removed to prevent distortion by extreme values. The effects of water treatments on changes in box weights were evaluated using a repeated-measures analysis of variance (ANOVA) model (data were natural log transformed), with monitoring occasion set as a repeated categorical variable. Treatment group, monitoring occasion and their interaction were set as fixed explanatory variables. Selection of repeated covariate type (autoregressive, unstructured, compound symmetry or toeplitz) was based on AIC of the full model. Again, model comparisons were made using ML, and final vales were obtained using REML. All statistical analyses were conducted using SAS version 9.3 (SAS Institute Inc., Cary, USA).

## References

[1] Field, C. B. *et al*. Climate Change 2014: Impacts, Adaptation, and Vulnerability. Part A: Global and Sectoral Aspects. Contribution of Working Group II to the Fifth Assessment Report of the Intergovernmental Panel on Climate Change (Cambridge University Press, 2014).

[2] Allen CD (2010). A global overview of drought and heat induced tree mortality reveals emerging climate change risks for forests. Forest Ecol. Manag..

[3] Wingfield MJ, Brockerhoff EG, Wingfield BD (2015). & Slippers, B. Planted forest health: the need for a global strategy. Science.

[4] Ghelardini L, Pepori AL, Luchi N, Capretti P, Santini A (2016). Drivers of emerging fungal diseases of forest trees. Forest Ecol. Manag..

[5] Ayres MP, Lombardero MJ (2000). Assessing the consequences of global change for forest disturbance from herbivores and pathogens. Sci. Total Environ..

[6] Finnish Statistical Yearbook of Forestry 2014 (ed. Peltola, A.) 317–372. (Tammerprint Oy, 2014).

[7] Schlyter P, Stjernquist I, Bärring L, Jönsson AM, Nilsson C (2006). Assessment of the impacts of climate change and weather extremes on boreal forests in northern Europe, focusing on Norway spruce. Climate Res..

[8] Kellomäki S, Peltola H, Nuutinen T, Korhonen KT, Strandman H (2008). Sensitivity of managed boreal forests in Finland to climate change, with implications for adaptive management. Philos. T. R. Soc. B.

[9] Lévesque M (2013). Drought response of five conifer species under contrasting water availability suggests high vulnerability of Norway spruce and European larch. Global Change Biol..

[10] Gauthier S, Bernier P, Kuuluvainen T, Shividenko AZ, Schepaschenko DG (2015). Boreal forest health and global change. Science.

[11] Christiansen E, Horntvedt R (1983). Combined *Ips/Ceratocystis* attack on Norway spruce, and defense mechanisms of the trees. J. Appl. Entomol..

[12] Krokene P, Solheim H (1998). Assessing the virulence of four bark beetle-associated bluestain fungi using Norway spruce seedlings. Plant Pathol..

[13] Repe A, Bojović S, Jurc M (2015). Pathogenicity of ophiostomatoid fungi on *Picea abies* in Slovenia. Forest Pathol..

[14] Björkman, C. & Niemelä, P. (eds). Climate Change and Insect Pests (CABI, 2015).

[15] Økland, B., Nethener, S. & Marini, L. *The Eurasian spruce bark beetle: The role of climate* in Climate Change and Insect Pests (eds Björkman, C. & Niemelä, P.) 202–219 (CABI, 2015).

[16] Økland B, Christiansen E (2001). Analysis of data from large-scale trapping of *Ips typographus* 1979–2000. Aktuelt fra Skogforsk.

[17] Netherer S (2015). Do water-limiting conditions predispose Norway spruce to bark beetle attack?. New Phytologist.

[18] Nagy NE (2004). Induced responses to pathogen infection in Norway spruce phloem: changes in polyphenolic parenchyma cells, chalcone synthase transcript levels and peroxidase activity. Tree Physiol..

[19] Zhao T (2011). Induced terpene accumulation in Norway spruce inhibits bark beetle colonization in a dose-dependent manner. PLoS ONE.

[20] Hammerbacher A (2013). A common fungal associate of the spruce bark beetle metabolizes the stilbene defences of Norway spruce. Plant Physiol..

[21] Wadke N (2016). The bark beetle-associated fungus, *Endoconidiophora polonica*, utilizes the phenolic defense compounds of its host as a carbon source. Plant Physiol..

[22] Jactel, H. *et al*. Drought effects on damage by forest insects and pathogens: a meta-analysis. *Global Change Biol.* **18**, 267–276 (2012).

[23] Christiansen E, Glosli A-M (1996). Mild drought enhances the resistance of Norway spruce to a bark beetle-transmitted blue-stain fungus. USDA, Forest Service. Gen. Tech. Report NC.

[24] Netherer S, Ehn M, Blackwell E, Kirisits T (2016). Defence reactions of mature Norway spruce (*Picea abies*) before and after inoculation of the blue-stain fungus *Endoconidiophora polonica* in a drought stress experiment. Forestry J..

[25] Linnakoski, R., Forbes, K. M., Wingfield, M. J., Pulkkinen, P. & Asiegbu, F. O. Testing projected climate change conditions on the *E**ndoconidiophora polonica*/Norway spruce pathosystem shows fungal strain specific effects. *F**ront. Plant Sci*. **8**, 883 (2017). 10.3389/fpls.2017.00883 PMC5445173 28603538

[26] Sallé A (2005). Fungal flora associated with *Ips typographus*: frequency, virulence, and ability to stimulate the host defence reaction in relation to insect population levels. Can. J. Forest Res..

[27] Boden S, Kahle H-P, von Wilper K, Spiecker H (2014). Resilience of Norway spruce (*Picea abies* (L.) Karst) growth to changing climatic conditions. Forest Ecol. Manag..

[28] Berryman AA (1972). Resistance of conifers to invasion by bark beetle–fungus associations. BioScience.

[29] Walters D, Heil M (2007). Costs and trade-offs associated with induced resistance. Physiol. Mol. Plant P..

[30] Bolton MD (2009). Primary metabolism and plant defense–fuel for the fire. Mol. Plant Microbe In..

[31] Linnakoski R (2016). Seasonal succession of fungi associated with *Ips typographus* beetles and their phoretic mites in an outbreak region of Finland. PLoS ONE.

[32] Jankowiak R (2006). Fungi associated with *Tomicus piniperda* in Poland and assessment of their virulence using Scots pine seedlings. Ann. Forest Sci..

[33] Littell, R. C., Milliken, G. A. Stroup, W. W. & Wolfinger, R. D. *SAS system for mixed models* (SAS Institute, Cary, 2006).

